# Low-Frequency Noise of Magnetic Sensors Based on the Anomalous Hall Effect in Fe–Pt Alloys

**DOI:** 10.3390/s19163537

**Published:** 2019-08-13

**Authors:** Yiou Zhang, Qiang Hao, Gang Xiao

**Affiliations:** Department of Physics, Brown University, Providence, RI 02912, USA

**Keywords:** anomalous Hall effect, noise measurement, Fe–Pt alloy

## Abstract

We took advantage of the large anomalous Hall effect (AHE) in Fe–Pt ferromagnetic alloys and fabricated magnetic sensors for low-frequency applications. We characterized the low-frequency electronic noise and the field detectability of the Fe_x_Pt_100-x_ system with various thin film thicknesses and Fe concentrations. The noise source consisted of 1/*f* and Johnson noise. A large current density increased the 1/*f* noise but not the Johnson noise. We found that the field detectability of the optimized Fe–Pt thin film offers much better low-frequency performance than a highly sensitive commercial semiconductor Hall sensor. Anomalous Hall effect sensors are, therefore, good candidates for magnetic sensing applications.

## 1. Introduction

The anomalous Hall effect (AHE) in ferromagnetic metals and alloys has drawn a great deal of attention as a potential candidate for magnetic field sensing applications [1,2,3,4,5,6,7,8,9]. Due to its strong spin–orbit interaction (SOI), Fe–Pt alloys exhibit some of the largest AHE among all ferromagnetic metals [3,10,11,12,13]. Compared with widely used semiconductor Hall effect sensors [14,15,16], Fe–Pt alloys are stable and easy to fabricate using a typical thin-film fabrication process. The metallic nature of AHE sensors also allows broader frequency response up to multiple GHz [6]. While sensitivity of an AHE sensor may not be as high as a semiconductor Hall sensor, characterization and comparison of noise properties are essential for a fair comparison. Nevertheless, there have been little if any studies on the intrinsic electronic noise behavior of the AHE sensors and its comparison with the traditional semiconductor Hall sensors. In order to comprehensively characterize the performance and capability of the AHE sensor, we conducted a systematic measurement of its noise spectra and sensitivity to reveal its intrinsic sensing capability. In particular, we focused on Fe_x_Pt_100-x_ thin-film alloys with various thicknesses and Fe atomic concentrations.

## 2. Materials and Methods

We prepared the Fe_x_Pt_100-x_ thin films using the high vacuum magnetron sputtering technique which is detailed in [10]. We patterned the films into Hall bars with a single step lift-off photo-lithography process. All measurements were performed at room temperature. We used the standard four-probe method to measure AHE resistivity under an out-of-plane magnetic field [11]. We measured the noise spectra from the Hall leads using the two-channel time cross-correlation method [17]. All noise measurement was performed over a broad frequency range from 1 Hz to 5 kHz. From the measured field sensitivity and noise spectrum, we calculated the field detectability ($S_{T}$, in unit of $T^{2}/Hz$), defined as the noise spectral density ($S_{V}$, in unit of $V^{2}/Hz$) divided by sensitivity, under a specific input (or measuring) current into the AHE sensor.

## 3. Results and Discussion

**Figure 1:**
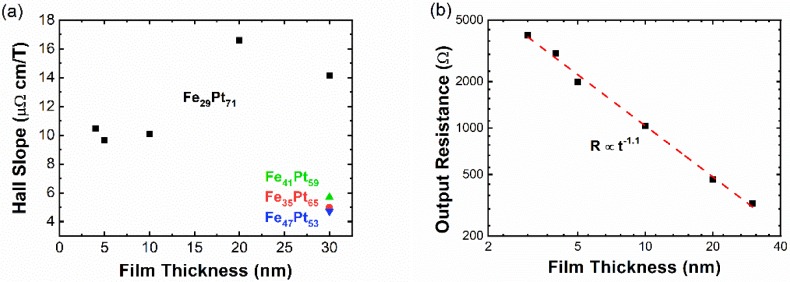
(**a**) Hall slopes versus film thickness and Fe concentration x. (**b**) Output resistance of Fe_29_Pt_71_ Hall-bar samples with various film thicknesses. The red dashed line is the linear fitting line in the log–log plot, which gives $R\sim t^{-1.1}$.

The AHE resistivities of all our Fe_x_Pt_100-x_ thin-film samples were found to be linear in the magnetic field up to the saturation fields (4π*M_s_*, where *M_s_* is the saturation magnetization). The results were presented in our previous work [3,10,11]. We also found that both intrinsic (Berry phase) mechanism and extrinsic side-jump mechanism contribute to the AHE, regardless of Fe concentration [10]. We first investigated the effect of Fe concentration x in the Fe_x_Pt_100-x_ thin film. As shown in Figure 1a, x = 29 gives a much higher Hall slope than other Fe concentrations. As for thickness dependence, we obtained the highest Hall slope of 16.6 µΩ·cm/T in the 20 nm thick Fe_29_Pt_71_ sample at room temperature. Correspondingly, the best field sensitivity reaches 23.6 V/A T, which is smaller than the field sensitivity of traditional semiconductor Hall sensors [14,16]. Another important parameter is the output resistance of our Hall sensor, which is the resistance between the two Hall leads. As discussed later, the output resistance defines the noise floor at high frequency. Additionally, low output resistance is required for radio-frequency application. Figure 1b shows output resistance for the Fe_29_Pt_71_ samples with various thicknesses. The output resistance follows a power-law relationship as $R\sim t^{-1.1}$ (the exponent is close to 1, as would be expected).

**Figure 2:**
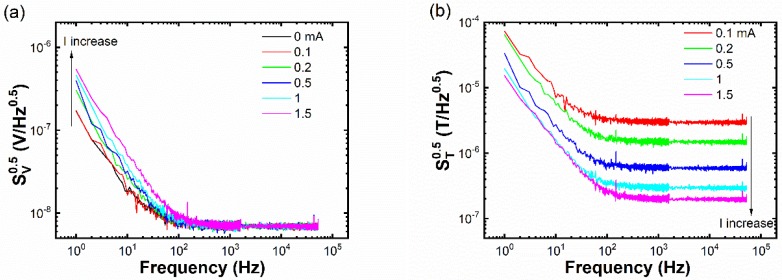
(**a**) Noise spectra and (**b**) field detectability spectra of the 4 nm thick Fe_29_Pt_71_ thin-film sample under various input currents.

Figure 2a shows some noise spectra of a representative 4 nm thick Fe_29_Pt_71_ thin-film sample under various input currents from 0 to 1.5 mA. At high frequency, the white Johnson noise dominates and shows no dependence on the input current. On the other hand, the low-frequency 1/*f* noise tends to increase as the input current rises above 0.1 mA. The knee frequency $f_{knee}$ can be defined as the crossover point between 1/*f* noise and white noise, where 1/*f* noise equals Johnson noise. Spectra of the field detectability are shown in Figure 2b. At high frequency, a larger input current leads to better detectability. The effect of input current becomes complicated at low frequency. At relatively small input current, a larger input current improves field detectability. At a large input current, low-frequency field detectability becomes almost independent of input current.

**Figure 3:**
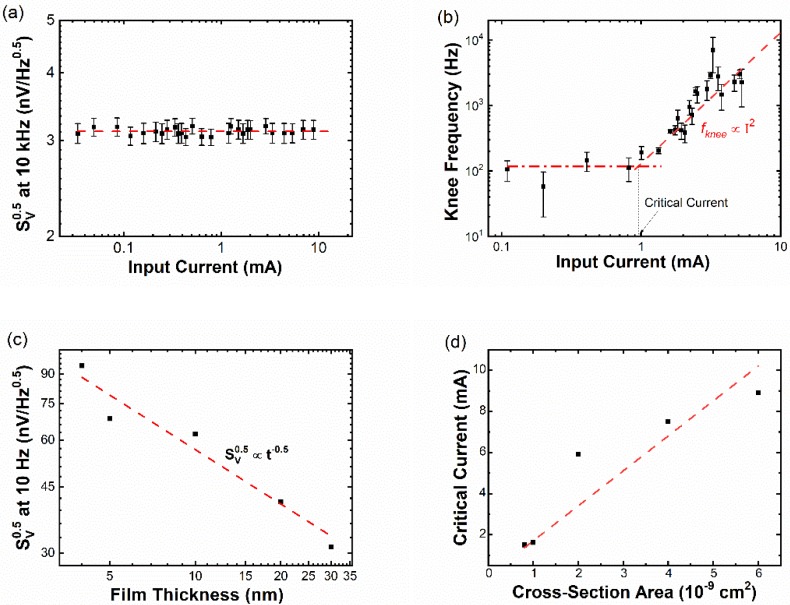
(**a**) High-frequency noise of the 20 nm thick Fe_29_Pt_71_ sample under various input currents. The red dashed line shows the theoretical prediction of Johnson noise $\sqrt{4k_{B}RT}$. (**b**) Knee frequency of the Fe_29_Pt_71_ sample under various input currents. Above critical current (~1 mA), knee frequency increases quadratically with input current. (**c**) Low-frequency noise of the Fe_29_Pt_71_ sample with different thicknesses. Input current is kept below critical current. The red dashed line shows the linear fitting in the log–log plot, which gives $S_{v}^{1/2}\sim t^{-0.5}$. (**d**) Critical current of the Fe_29_Pt_71_ sample with different cross-section areas (width of the Hall bar is 20 μm). The slope of the red dashed line gives the critical current density of $1.7\times 10^{6}\;A/{\mathrm{cm}}^{2}$.

To understand such behavior, we measured noise spectra of a 20 nm thick Fe_29_Pt_71_ sample under a broad range of input currents (0.01 to 8.9 mA). As shown in Figure 3a, high-frequency noise is independent of input current, and its value can be well explained by Johnson noise. As long as the input current is not large enough to significantly heat up the thin film, high-frequency white noise is unchanged. Since high-frequency white noise is unchanged, we can use knee frequency to characterize low-frequency noise. Figure 3b shows $f_{knee}$ at different input currents for the 5 nm thick Fe_29_Pt_71_ sample. When the input current is less than 1 mA, $f_{knee}$ is nearly constant. However, beyond 1 mA, $f_{knee}$ increases quadratically with input current. The transition point between low and high input current is defined as the critical current. Figure 3c shows the low-frequency noise at 10 Hz of the Fe_29_Pt_71_ samples with different thicknesses below each sample’s critical current. 1/*f* noise is commonly believed to be the thermal fluctuation of discrete fluctuators. The noise power of 1/*f* noise is inversely proportional to the number of fluctuators. Therefore, the power-law relationship ($S_{v}^{1/2}\sim t^{-0.5}$) is expected, assuming that the density of fluctuators has no dependence on film thickness. Figure 3d shows the relationship between critical current and sample cross-section area. As expected, a linear relationship is observed, and the slope gives a critical current density (*J_c_*) of $1.7\times 10^{6}\;A/{\mathrm{cm}}^{3}$. This number is an intrinsic value of the AHE sensor at a particular Fe concentration x. Deviation of the data from the fitting line is mainly due to uncertainty in determining the critical current. In addition, a small offset on the *x*-axis can be observed, which can be attributed to the surface dead layer effect [18]. The desired input current is slightly higher than the critical current. Thus, low-frequency detectability is optimized and power consumption of the AHE sensor is not too large.

**Figure 4:**
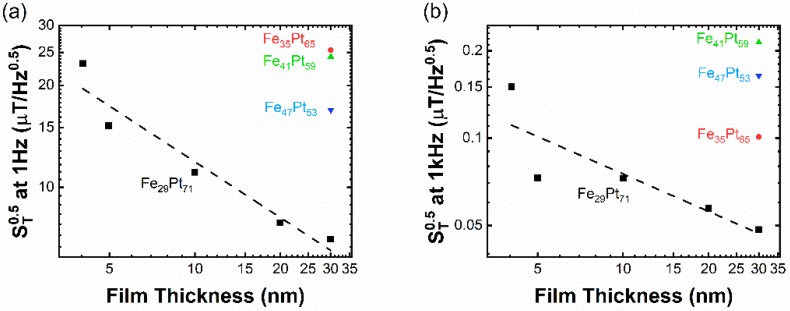
Field detectability of different Fe_x_Pt_100-x_ sensors at (**a**) 1 Hz and (**b**) 1 kHz. Both low-frequency and high-frequency detectabilities show the power-law relationship with film thickness, with the exponent close to −0.5.

Figure 4a,b shows the field detectability of the Fe_x_Pt_100-x_ samples at high and low frequencies. For the fixed sample thickness, the Fe_29_Pt_71_ alloy has the largest Hall slope and the best detectability value. At both high and low frequencies, field detectability follows a power-law relationship with film thickness, with the exponent close to −0.5. The best detectability is achieved in the 30 nm thick Fe_29_Pt_71_ sample ($50\;nT/\sqrt{Hz}$ at 1 kHz and $7\mu T/\sqrt{Hz}$ at 1 Hz).

**Figure 5:**
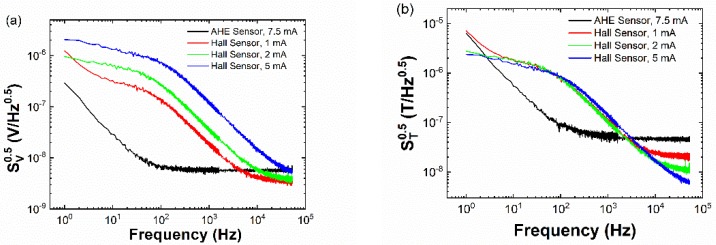
Comparison of the (**a**) voltage noise spectra and (**b**) field detectability spectra between the 30 nm thick Fe_29_Pt_71_ anomalous Hall effect (AHE) sensor and a commercial semiconductor Hall sensor.

For comparison in performance, we measured the voltage noise and field detectability spectra of a highly sensitive commercial semiconductor Hall sensor acquired from LakeShore (Model HGT-2101, Westerville, OH, USA). Commercial Hall sensors typically suffer from random telegraph noise (RTN) [19]. Figure 5a,b shows the comparison in noise behavior between the commercial Hall sensor and the AHE sensor. As can be seen, the low-frequency noise of the AHE sensors is one to two orders of magnitude smaller than that of the semiconductor Hall sensor. Even though the sensitivity of our AHE thin-film sensor (8.2 V/A T) is one order of magnitude lower than that of the semiconductor Hall sensor (173 V/A T), the AHE sensor outperforms the semiconductor Hall sensor in terms of field detectability in the frequency range of 3 to 1500 Hz. Similar to 1/*f* noise, the noise power of RTN is inversely proportional to the number of fluctuators. Roughly speaking, the number of fluctuators is related to the number of charge carriers. Therefore, the low carrier density of semiconductor Hall sensors leads to larger noise at low frequency, which compensates for the high sensitivity. On the other hand, AHE sensors have a much higher carrier density and thus reduced low-frequency noise. Since low-frequency noise of both sensors scales with input current, an increase in input current does not improve their low-frequency performance. On the other hand, both sensors should have better field detectability at high frequency if input current is further increased.

## 4. Conclusions

In conclusion, we characterized the noise behavior and the magnetic sensing capability of anomalous Hall effect sensors based on Fe_x_Pt_100-x_ thin-film alloys with variable thicknesses and Fe concentration x. In the Fe_x_Pt_100-x_ system, the field detectability depends on sample thickness, Fe concentration x, Hall slope, and input (measuring) current density. Fe_29_Pt_71_ thin films offer the best field detectability, that is, $50\;nT/\sqrt{Hz}$ at 1 kHz and $7\;\mu T/\sqrt{Hz}$ at 1 Hz. The Fe_29_Pt_71_ AHE sensor outperforms a highly sensitive commercial Hall sensor in the frequency range of 31–500 Hz. The AHE sensor is metal based and can be easily fabricated. Its low-frequency magnetic sensing performance makes it a promising magnetic sensor candidate. Further optimization in AHE sensors may make AHE sensors rival the best semiconductor Hall sensors.
